# TILDA-X: Transcriptome-Informed Lung Cancer Disparities via Explainable AI

**DOI:** 10.3390/cancers17213454

**Published:** 2025-10-28

**Authors:** Masrur Sobhan, Md Mezbahul Islam, Mary Jo Trepka, Gregory E. Holt, Charles J. Dimitroff, Ananda M. Mondal

**Affiliations:** 1 Machine Learning and Data Analytics Group (MLDAG), Knight Foundation School of Computing and Information Sciences, Florida International University, Miami, FL 33199, USA; msobh002@fiu.edu (M.S.); misla093@fiu.edu (M.M.I.); 2 Department of Epidemiology, Robert Stempel College of Public Health & Social Work, Florida International University, Miami, FL 33199, USA; trepkam@fiu.edu; 3 Department of Medicine, University of Miami, Coral Gables, FL 33146, USA; gholt@med.miami.edu; 4 Department of Cellular and Molecular Medicine, Herbert Wertheim College of Medicine, Florida International University, Miami, FL 33199, USA; cdimitroff@fiu.edu

**Keywords:** explainable AI, health disparity, lung cancer, patient-specific biomarker, SHAP

## Abstract

**Simple Summary:**

This research addresses the challenge of unequal outcomes in lung cancer across racial and sex groups. Traditional approaches that classify patients by race often produce biased results due to imbalanced data. To overcome this, the authors created a new framework called TILDA-X, which uses disease conditions instead of race for classification and employs explainable artificial intelligence to identify meaningful biomarkers. By discovering individual patient biomarkers first and then building up to group-level patterns, the method uncovers unique biological pathways linked to disparities between different racial and sex groups. The results show that this approach is far more accurate and biologically valid than race-based classification. These findings provide a robust and more reliable way to understand lung cancer differences, supporting the development of precision medicine that can benefit diverse patient populations.

**Abstract:**

Background: Lung cancer is a leading cause of cancer-related mortality, with disparities in incidence and outcomes observed across different racial and sex groups. Identifying both patient-specific and cohort-specific disparity biomarkers is critical for developing targeted treatments. The lung cancer dataset is highly imbalanced across races, leading to biased results in disparity information if classification is based on race. Method: This study developed an explainable artificial intelligence-based framework, TILDA-X, which designs classification models based on disease conditions instead of races to mitigate racial imbalance in the dataset and applies explainable AI to delineate patient-specific disparity information. A lung cancer transcriptome dataset with three disease conditions—lung adenocarcinoma, lung squamous cell carcinoma, and healthy samples—was used to develop classification models. Applying a bottom-up approach from patient-specific disparity information, the cohort-specific disparity information is discovered for different racial and sex groups, African American males, European American males, African American females, and European American females. Results: Classification based on disease conditions achieved accuracy between 88% and 100% for minority groups (African American males and females), whereas it was only between 0% and 16% for race-based classification, which underscores the significance of the proposed approach. Functional analysis of sub-cohort-specific biomarker genes revealed unique pathways associated with lung cancers in different races and sexes. Among the significant pathways identified, over ~63% overlapped with previously reported lung cancer-related studies, supporting the biological validity of our findings. Overall, combining disease conditions-based classification with explainable AI, this study provides a robust, interpretable framework for characterizing race- and sex-specific disparities in lung cancer, offering a foundation for precision oncology and equitable therapeutic development based on transcriptome profile only.

## 1. Introduction

In the United States, lung cancer is the leading cause of cancer deaths. In 2023, approximately 350 deaths per day occurred from lung cancer [1], 81% of which were caused by cigarette smoking directly, with an additional 3% due to second-hand smoke [2]. The estimated new case (75 vs. 67 in 100,000) and death rates (51 vs. 45 in 100,000) in lung cancer are higher among African American Males (AAMs) than in European American Males (EAMs) [1]. On the other hand, the estimated new case (47 vs. 56 in 100,000) and death rates (28 vs. 33 in 100,000) are lower among African American Females (AAFs) than European American Females (EAFs) [1]. Thus, there exists a complex disparity puzzle in lung cancer etiology between African Americans (AAs) and European Americans (EAs) in terms of both race and sex.

Globally, lung cancer remains one of the most prevalent and lethal malignancies, accounting for approximately 11.4% of all new cancer cases and nearly 18% of cancer-related deaths worldwide [3]. The global data also exhibits clear geographic and demographic variation. East Asia reports the highest incidence of lung adenocarcinoma, while Eastern Europe shows elevated rates of squamous cell carcinoma, reflecting differences in environmental exposure, smoking prevalence, and genetic susceptibility [4,5,6,7,8]. These global patterns emphasize that lung cancer heterogeneity is shaped not only by environmental exposures, but also by population-specific biological and molecular factors.

Cigarette smoking is considered the strongest risk factor for lung cancer, but smoking alone cannot explain the disparity of lung cancer development between AAs and EAs [9]. Based on genome-wide association studies (GWAS) for 13 cancers, Sampson et al. [10] found that only 24% of lung cancer’s heritability can be attributed to genetic determinants of smoking, which indicates the complex nature of heterogeneity in lung cancer development, leading to health disparities. In a recent differential gene expression analysis (DGEA) using mRNA and miRNA expression profiles between lung tumors and normal adjacent to tumors (NAT) of AAs and EAs, researchers discovered ~3500 differentially expressed probes from AAs and ~4700 differentially expressed probes from EAs [9]. Many probes were common, along with 637 AA-specific and 1844 EA-specific probes. Surprisingly, principal component analysis showed that AA-specific differentially expressed probes could separate lung tumors and NAT samples in both AAs and EAs. This observation suggests that AA-specific differentially expressed probes/genes discovered using DGEA analysis cannot be considered AA-specific risk factors. The recent genome-wide association studies (GWAS), considering a large cohort of cases and controls for African Americans (AAs) [11] and European Americans (EAs) [12], failed to discover AA-specific susceptible loci since both studies discovered the same two loci near plausible candidate genes, *CHRNA5* and *TERT*, on 15q25 and 5p15, respectively, associated with lung cancer risk in both AAs and EAs.

Recent studies have revealed that both genetic ancestry and sex-specific biology profoundly shape tumor behavior, immune regulation, and clinical outcomes. Population-based analyses show that AA patients tend to present with more aggressive tumor features, higher genomic instability, and altered immune profiles compared to EA patients, even after controlling for socioeconomic and environmental factors [9]. Transcriptomic and methylation-based studies have reported distinct patterns of gene regulation in AAs, including the differential expression of DNA repair genes, oncogenic signaling mediators, and immune-related genes, all of which contribute to tumor progression and therapeutic resistance [13]. May et al. highlight men generally experience higher lung cancer mortality and more aggressive disease, while women show better responses to certain chemotherapies and targeted therapies emphasizing that these differences arise from complex interactions between sex hormones (estrogen, progesterone, testosterone), genetic and immune variations, and environmental exposures such as smoking [14]. From an immunogenomic perspective, African American patients with non-small cell lung cancer (NSCLC) often exhibit enhanced interferon and inflammatory signaling and increased cytotoxic T-cell infiltration, yet experience poorer clinical outcomes, reflecting an imbalance between immune activation and suppression [15]. Moreover, sex-based differences in gene expression and hormonal signaling—particularly involving estrogen receptor, FOXA1, and immune checkpoint pathways—further influence lung tumor progression and treatment outcomes [15,16,17,18,19]. However, most genomic studies to date have not systematically disentangled the interacting effects of race, sex, and tumor subtype, underscoring the need for integrative frameworks that can parse these overlapping biological influences at the individual and sub-cohort level.

Feature selection has long been a valuable approach for identifying biomarker genes in various cancers [20,21,22]. Several studies have focused on discovering lung cancer biomarkers using machine learning and deep learning approaches. For example, Sobhan et al. employed a deep learning-based feature selection algorithm to identify key genes that can differentiate lung cancers between AAMs and EAMs, revealing molecular patterns linked to racial disparities [23]. Additionally, researchers have explored the use of explainable machine learning techniques, such as SHAP (SHapley Additive ExPlanations) [24], to identify patient-specific biomarker genes in lung cancer patients [25]. While this research effectively identified patient-specific biomarkers for two types of lung cancer, including lung adenocarcinoma (LUAD) and lung squamous cell carcinoma (LUSC) patients, it did not address the race- and sex-specific lung cancer health disparity.

Recent advances in machine learning (ML) and deep learning (DL) have accelerated the discovery of prognostic and molecular features across diverse populations, improving our understanding of tumor heterogeneity and clinical outcomes. For instance, Nicolas et al. integrated transcriptomic and radiomic clinical, pathological, radiological, and transcriptomic data using machine learning to predict immunotherapy response in NSCLC, emphasizing the importance of machine leaning models in clinical translation [26]. Similarly, Seema et al. developed an attention-based multi-omics integration model to identify sub-cohort-specific molecular drivers in NSCLC [27]. Deep learning frameworks such as graph attention networks (GATs) [28,29] and variational autoencoders (VAEs) [30] have also been applied to multi-omics data for patient stratification and biomarker discovery.

However, despite these promising advances, existing research on lung cancer disparities rarely incorporates both race and sex dimensions simultaneously. Most studies are based on AAs and EAs, meaning the cohort is a male and female mix. DGEA and GWAS’s inability to discover AA-specific risk loci is because these approaches are cohort-based and largely ignore the genetic and epigenetic variability of individuals or intratumor heterogeneity (ITH), and resulted in population-based conclusions or one-size-fits-all solutions [31]. A recent committee on “Using Population Descriptors in Genetics and Genomics Research” concluded that there is no one-size-fits-all solution since research conducted using genomics data is broad and varied [32]. Thus, there is an overarching need for an alternative approach to discover risk factors that can explain the lung cancer health disparity not only between AAs vs. EAs, but also between AAMs, EAMs, AAFs, and EAFs. To address the limitations of traditional cohort-based methods in disentangling the underlying sources of health disparities, we developed a computational framework, TILDA-X (Transcriptome-Informed Lung Cancer DispArities via EXplainable AI), to discover and interpret the lung cancer health disparity by leveraging machine learning and the explainable AI approach, SHAP [24]. To address the race-specific data imbalance, we designed the classification task based on disease conditions (LUAD, LUSC, and HEALTHY) instead of race or sex.

To explicitly highlight the novelty and contributions of this study, we emphasize that previous works on lung cancer health disparities have primarily focused on cohort-level analyses (e.g., GWAS, DGEA), which fail to capture individual-level molecular heterogeneity and the combined effects of race and sex. In contrast, our proposed framework introduces an explainable AI-based bottom-up strategy that deciphers disparities at the patient and sub-cohort levels. Unlike traditional classification approaches that use race or sex as class labels, this framework adopts a disease-based classification (LUAD, LUSC, and HEALTHY), thereby overcoming race-specific data imbalance and ensuring unbiased learning. By leveraging SHAP-based interpretability, the framework provides biologically meaningful insights into patient-specific and cohort-specific biomarker genes associated with racial and sex disparities. The salient features and contributions of this study are enumerated below.

- We assume that the local interpretation of SHAP or the interpretation of each patient of AA and EA cohorts under different disease classes/labels, such as LUAD, LUSC, and HEALTHY, will help extract patient-specific significant genes reflecting patient-specific disparity related to those disease conditions.
- We also assume that the interpretation of a patient using the different combinations of disease classes (LUAD-LUSC-HEALTHY; LUAD-LUSC; LUAD-HEALTHY; LUSC-HEALTHY) would help derive a robust set of patient-specific genes. Each of the LUAD patients (irrespective of race and sex) was interrogated via three classification problems containing the LUAD cohort, namely LUAD-LUSC-HEALTHY, LUAD-LUSC, and LUAD-HEALTHY. In a similar way, each LUSC patient was interrogated via three classification problems containing the LUSC cohort (LUAD-LUSC-HEALTHY, LUAD-LUSC, and LUSC-HEALTHY).
- We explored SHAP in a bottom-up approach (going from patient-specific biomarker genes to cohort-specific biomarker genes) to decipher the disparity between any two cohorts of patients, including AAMs vs. AAFs, AAMs vs. EAMs, AAMs vs. EAFs, AAFs vs. EAMs, AAFs vs. EAFs, and EAMs vs. EAFs.
- Note that the classification problems are designed based on disease conditions (i.e., LUAD, LUSC, and HEALTHY) to avoid the race-specific imbalance in the dataset, which is innovative in discovering the health disparity. The data is highly imbalanced regarding race (AA:EA = 1:8). But input to the SHAP is a classification problem with disease conditions LUAD (*n* = 356), LUSC (*n* = 295), and HEALTHY (*n* = 313) as class labels, meaning the data is balanced (each class consists of ~300 samples). The race-specific high accuracies (AAM: 90% and EAM: 95%) for an imbalanced cohort ratio (AAM:EAM = 1:7) support our hypothesis.

Since the transcriptome mirrors both genomic and epigenomic variability or ITH [33], this study interrogated individual patients via classification problems designed using transcriptome or expression profiles of ~20,000 genes. To our knowledge, no prior study has used SHAP or other XAI frameworks to analyze gene-level contributions across race- and sex-stratified lung cancer cohorts at an individual patient level. Our work uniquely integrates explainable machine learning with health disparity research, allowing us to identify patient-specific and cohort-specific biomarker patterns at the race and sex level. This bottom-up approach reveals differences not just between race and sex sub-cohorts (e.g., AAMs vs. AAFs or AAMs vs. EAMs) but also within broad groups (e.g., AAs vs. EAs), providing a more comprehensive understanding of race and sex disparities in lung cancer.

## 2. Materials and Methods

### 2.1. Data Collection

RNA-seq gene expression data for LUAD, LUSC, and HEALTHY tissues were downloaded from the publicly available memorial sloan kettering cancer center (MSKCC) GitHub repository (https://github.com/mskcc/RNAseqDB, accessed on 1 August 2024) [34]. Originally, the disease samples were sourced from The Cancer Genome Atlas (TCGA) [35] and the healthy control samples from the Genotype-Tissue Expression (GTEx) project [36]. Typically, RNA-seq data from different studies are not directly comparable due to differences in sample processing, data normalization, and potential batch effects. However, the MSKCC repository provides pre-processed data where such biases have been corrected, enabling reliable comparative analysis across the TCGA and GTEx datasets. The analysis utilized normalized FPKM (Fragments Per Kilobase of transcript per Million mapped reads) gene expression data.

### 2.2. Data Preparation

The initial dataset comprised 503 LUAD, 489 LUSC, and 423 HEALTHY samples, totaling 1415 samples. After removing duplicate entries by retaining only the first instance of each duplicate ID, the dataset was reduced to 1401 samples. The HEALTHY cohort included samples from GTEx healthy tissues and normal adjacent to tumor (NAT) samples from LUAD and LUSC cases. However, for this study, NAT samples were excluded, resulting in 313 HEALTHY samples. To further refine the dataset, we included only samples belonging to the ‘non-Hispanic or Latino’ ethnic group and excluded any samples lacking race or sex information. This filtering process yielded a final dataset of 964 samples, as shown in Table 1, with race- and sex-specific breakdown. Note that the GTEx data do not have race and sex information, and it is not an issue for this study since the classification problems were designed based on disease conditions (LUAD, LUSC, and HEALTHY). The gene expression data consisted of 19,648 genes or features, which were utilized for subsequent analysis. The final dataset, as shown in Table 1, was used to classify three classes—LUAD, LUSC, and HEALTHY—using various machine learning algorithms.

### 2.3. Study Flow Diagram

**Figure 1:**
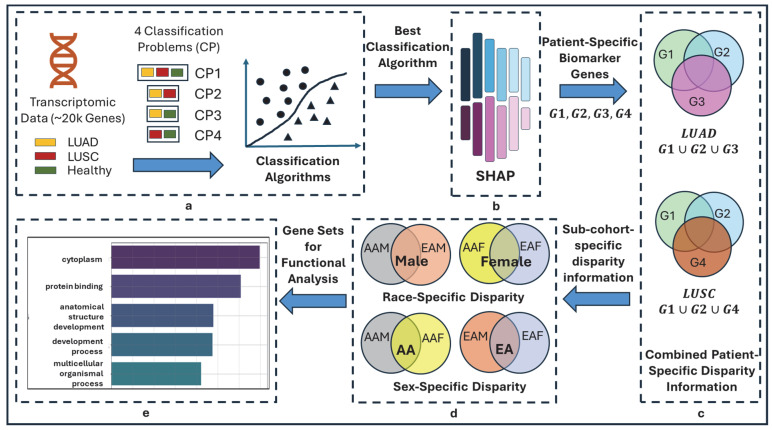
Overall flow diagram of TILDA-X. (**a**) Multiple classification problems (CPs). CP1: 3-class CP with labels LUAD, LUSC, and HEALTHY; CP2: 2-class CP with labels LUAD and LUSC; CP3: 2-class CP with labels LUAD and HEALTHY; CP4: 2-class CP with labels LUSC and HEALTHY. (**b**) Local Feature Interpretation using SHAP. Output of SHAP is patient-specific biomarker gene sets-G1, G2, G3, and G4 from four classification problems—CP1, CP2, CP3, and CP4, respectively. Note that each of G1, G2, G3, and G4 will have different sets of genes for different patients. (**c**) Combined Patient-Specific Disparity Information. A LUAD patient belongs to three classification problems—CP1, CP2, and CP3. SHAP discovered three sets of patient-specific significant genes—G1, G2, and G3 (each set from each classification problem)—for each LUAD patient. Union of three sets of genes (G1∪G2∪G3) generates combined patient-specific disparity information. Similarly, (G1∪G2∪G4) generates combined patient-specific disparity information for LUSC. (**d**) Race- and sex-cohort-specific disparity information. (**e**) Validation.

The overall pipeline of this study is shown in Figure 1. The objective of this study is to delineate the race- and sex-specific health disparities between two cohorts (AAMs vs. AAFs, AAMs vs. EAMs, AAMs vs. EAFs, AAFs vs. EAMs, AAFs vs. EAFs, and EAMs vs. EAFs) in two types of lung cancers (LUAD and LUSC), leveraging machine learning algorithms and explainable AI, SHAP.

#### 2.3.1. Multiple Classification Problems and Rationale

In this study, we formulated four distinct classification problems using three class labels, including LUAD, LUSC, and HEALTHY, as shown in Figure 1a. One 3-class problem (LUAD-LUSC-HEALTHY) and three 2-class problems (LUAD-LUSC, LUAD-HEALTHY, and LUSC-HEALTHY). The rationale behind this approach is that each classification problem may highlight a different aspect of heterogeneity in lung cancer via the local interpretation of SHAP, thereby providing a more comprehensive understanding of lung cancer disparity. Consider a LUAD patient—In LUAD-LUSC-HEALTHY classification, SHAP will identify a set of patient-specific features (i.e., disparity information) that differentiate the patient of interest from both LUSC and HEALTHY patients; In LUAD-LUSC classification, SHAP will identify a second set of patient-specific features (i.e., disparity information) that differentiate the patient of interest from all LUSC patients; In LUAD-HEALTHY classification, SHAP will identify a third set of patient-specific features (i.e., disparity information) that differentiate the patient in interest from all HEALTHY patients. Thus, combining these three sets of patient-specific genes will provide comprehensive insights into lung cancer health disparities.

#### 2.3.2. Local Feature Interpretation Using SHAP

The SHAP is a powerful XAI tool based on game theory that helps us understand how machine learning models make decisions. SHAP assigns a score to each feature for every sample, showing how much each feature affects the model’s prediction. To calculate SHAP scores, it takes the machine learning model and the samples as input to observe how a specific feature changes the model’s prediction. It does this by comparing the model’s output with and without the feature of interest across different combinations of features, called coalition sets. The differences in predictions are calculated for each of these sets. By averaging these differences across all possible combinations, the SHAP score is calculated for a feature, which tells us how important that feature is for a particular prediction, also known as local feature interpretation.

In this study, SHAP was used to generate scores for all 964 samples across LUAD, LUSC, and HEALTHY groups, encompassing 19,648 genes or features. A higher SHAP score indicates greater importance of a feature. Subsequently, the genes for each patient were ranked in descending order based on their SHAP scores. The top 100 genes from this ranked list were selected, as these genes are believed to contain critical risk information for the patient and are therefore referred to as patient-specific biomarker genes that carry patient-specific disparity information.

#### 2.3.3. Combined Patient-Specific Biomarker Genes

Each LUAD sample was analyzed across three distinct classification tasks: LUAD-LUSC-HEALTHY, LUAD-LUSC, and LUAD-HEALTHY. We only considered the common correctly predicted samples from these three classification problems. As a result, each sample yielded three different sets of biomarker genes, corresponding to each classification. Next, by taking the union of these three gene sets, we obtain a comprehensive list of patient-specific biomarker genes that encapsulate the combined disparity information across all classification tasks. The same procedures were applied to LUSC and HEALTHY samples.

#### 2.3.4. Race- and Sex-Cohort-Specific Disparity Information

We used a bottom-up approach to determine the disparity information for the AAM sub-cohort by combining the lists of patient-specific genes within this sub-cohort. By aggregating these gene sets, we capture a wide spectrum of genetic markers that contribute to disparities in AAMs. The same approaches were conducted to identify the disparity-related genes in AAF, EAM, and EAF sub-cohorts.

Disparity among sub-cohorts: To show the disparity among sub-cohorts, we used upset plots showing the unique genes for each sub-cohort and common (intersected) genes among the sub-cohorts. The common or intersected genes represent the common characteristics among sub-cohorts. On the contrary, the unique genes of each sub-cohort show the unique genetic information of that group, which is different from others. These unique genes are assumed to bear genetic information which is causing the disparity among sub-cohorts.

Disparity between AA and EA cohorts: By following a bottom-up approach, the disparity information of the AA cohort will be discovered by combining the disparity information from sub-cohorts (AAMs and AAFs). Similarly, EA cohort disparity will come from EAMs and EAFs sub-cohorts.

#### 2.3.5. Validation

For validation, we performed functional enrichment analysis using g:Profiler, incorporating all available pathway databases. Pathways were considered significant only if they met the threshold of *p* < 0.05. To further validate the biological relevance of these findings, we conducted a comprehensive literature review to determine whether the identified significant pathways had been previously reported in lung cancer studies. The presence of substantial overlaps between our results and existing literature supports the credibility of our approach.

## 3. Results

### 3.1. Selecting the Best Machine Learning Approach

Six classification algorithms were employed in this study. These include fully connected neural networks (FCN), a deep learning approach; logistic regression (LR), a regression-based method; naïve Bayesian classifier (NB), a probabilistic model; support vector machine (SVM), a kernel-based method; and two tree-based methods, random forest (RF) and extreme gradient boosting (XGBoost). A five-fold cross-validation procedure was conducted to evaluate the performance of these classifiers. Initially, machine learning algorithms were implemented using their default hyperparameters (HPs) provided in the sklearn library for classifying LUAD, LUSC, and HEALTHY samples. Table 2 presents the classification performance (mean ± standard deviation) of six machine learning algorithms across four classification tasks using transcriptomic data.

Overall, XGBoost consistently outperforms other methods by achieving the highest accuracy in all tasks with minimal variability. This superior performance can be attributed to XGBoost’s ability to handle high-dimensional data, its robustness to multicollinearity, and its inherent regularization mechanisms that prevent overfitting, making it especially effective for complex biological data like gene expression. Fully connected neural networks also perform competitively but are slightly less stable, likely due to the small sample size relative to the number of features. Random forest also shows good performance, though slightly below XGBoost, with RF benefiting from ensemble learning but lacking the advanced boosting mechanisms of XGBoost. In contrast, NB and SVM perform poorly in LUAD-LUSC-HEALTHY, LUAD-HEALTHY, and LUSC-HEALTHY classification tasks. NB’s assumption of feature independence is violated in correlated gene expression profiles, while SVM struggles with high-dimensional, noisy data and non-linear class boundaries.

Next, we conducted hyperparameter tuning for the best-performing model, XGBoost, to improve classification accuracy across LUAD, LUSC, and HEALTHY classes. While tuning resulted in a very slight improvement in accuracy, it came at a significant computational cost. Given the time-intensive nature of tuning, especially when repeated across multiple experiments (seeds), we chose to proceed with the default hyperparameter settings for different runs of XGBoost classifiers to maintain computational efficiency without compromising result quality. To assess the robustness of our results, we ran each experiment ten times using different seeds, ranging from 10 to 100 (in increments of 10). This decision was guided by a prior study [37], which reported minor variations in performance due to changes in random initialization. Notably, in our case, XGBoost produced consistent results across all seeds, demonstrating the stability of the model. Considering both the negligible performance improvement from tuning and the consistent outcomes across seeds, using the default hyperparameters was a practical and justifiable choice.

### 3.2. Patient-Specific Disparity

**Figure 2:**
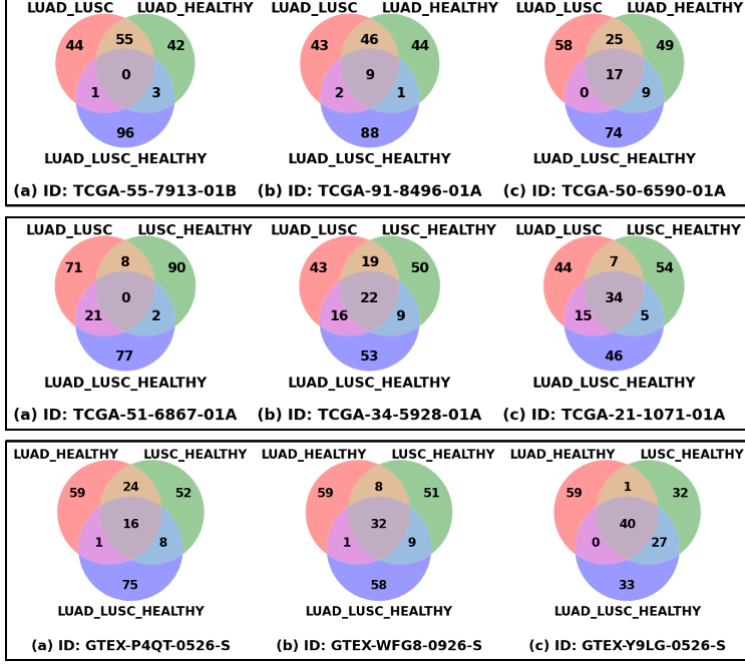
Patient-specific significant genes from three classification problems. Each Venn diagram represents the overlapping and unique top-ranked significant genes derived from three classification settings (LUAD_LUSC, LUSC_HEALTHY, and LUAD_LUSC_HEALTHY) based on SHAP score. Venn diagrams of three LUAD samples (Top Row), three LUSC samples (Middle Row), and three HEALTHY samples (Bottom Row) are shown in this figure. For each row: figure (**a**) represents the sample with minimum overlapping genes, figure (**b**) sample with median overlapping genes, and figure (**c**) sample with maximum overlapping genes.

Figure 2 shows the patient-specific significant genes reflecting patient-specific heterogeneity or disparity information. Each of the nine Venn diagrams represents three sets of the top 100 significant genes derived from three classification problems for a patient or healthy sample, which could be thought of as extracting the disparity information using three different types of interrogation. The union of these three sets of genes could be thought of as the representation of complete heterogeneity that exists in a patient or sample. The top row shows the Venn diagrams of three LUAD patients, the first one with the minimum number of common genes, the third one with the maximum number of common genes, and the middle one is an intermediate sample. In a similar way, the middle and bottom rows show the samples from the LUSC and HEALTHY cohorts. The ranges of common genes within the samples of LUAD, LUSC, and HEALTHY cohorts are 0 to 17, 0 to 34, and 16 to 40, respectively. The fewer common genes mean the samples are more heterogeneous, which is reflected in LUAD and LUSC cohorts, compared to HEALTHY samples, as expected.

### 3.3. Race- and Sex-Specific Correct Prediction

Table 3 shows the sub-cohort or race- and sex-specific correct prediction for the LUAD and LUSC cohorts. For example, the actual number of samples in the LUAD-AAM sub-cohort is 20, of which at least 18 samples were correctly predicted in all three classification problems in our proposed approach. This means that in some classifications, the correctly predicted number could be more than 18, and thus, 90% is the minimum of the three prediction accuracies. Similarly, LUAD-AAF has a minimum accuracy of 96%. To demonstrate the effectiveness of our proposed approach in accurately predicting samples, particularly from underrepresented sub-cohorts, we conducted two experiments: (i) a 4-class model, where the response variables are AAM, AAF, EAM, and EAF, within each disease category (LUAD and LUSC) independently; and (ii) an 8-class model to predict combined disease-demographic labels (LUAD-AAM, LUAD-AAF, LUAD-EAM, LUAD-EAF, LUSC-AAM, LUSC-AAF, LUSC-EAM, LUSC-EAF). The best performing model, XGBoost, was used for classification with default hyperparameters. In both experimental settings, we observed that the classifier exhibited bias toward majority classes, achieving only 0% to 16% accuracy for minority sub-cohorts such as AAM and AAF in both LUAD and LUSC. In contrast, our proposed approach demonstrated significantly improved performance in these imbalanced scenarios, yielding accuracy between 88% and 100% for the same underrepresented groups. These findings suggest that our method is more effective in addressing class imbalance and capturing sub-cohort-specific patterns. In other words, classification based on disease conditions removes the issue of racial imbalance in datasets.

While the sub-cohort sizes for AAMs and AAFs are relatively small, this limitation primarily reflects the unequal representation of racial groups in publicly available dataset. Importantly, our framework mitigates the potential effects of small sub-cohort sizes by employing a disease-based classification design instead of race, which balances the overall dataset and prevents the model from being biased toward major racial groups. Furthermore, we validated our model’s robustness through repeated multi-class experiments using different seed values, supporting the reproducibility of the findings.

### 3.4. Recovery of Gene Set-Level Disparity Within LUAD and LUSC Cohorts

**Figure 3:**
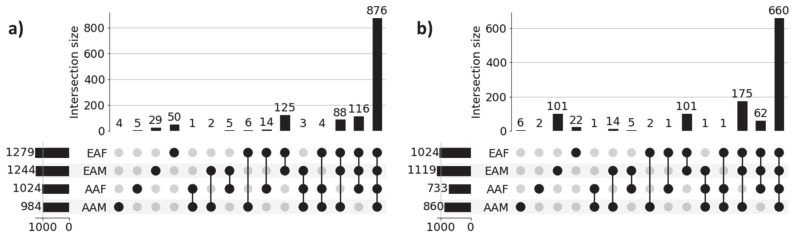
UpSet plots of sub-cohort-specific gene sets in (**a**) LUAD and (**b**) LUSC. Horizontal bars in the plot represent sub-cohort-specific significant genes. For LUAD, these numbers are—AAM: 984, AAF: 1024, EAM: 1244, and EAF: 1279. First 4 vertical bars represent the number of unique significant genes for each sub-cohort, and the remaining 11 bars represent the overlapping significant genes between sub-cohorts (AAM, AAF, EAM, and EAF). Most of the sub-cohort genes are common in both LUAD (876 genes) and LUSC (660 genes), indicating population-dependent transcriptomic signatures. Only a limited number of genes are unique to individual sub-cohorts indicating distinct transcriptomic signatures.

Figure 3 shows the UpSet plot diagrams of significant gene sets corresponding to the four sub-cohorts (AAM, AAF, EAM, and EAF) of LUAD and LUSC that illustrate the unique genes for each sub-cohort and intersections among the four sub-cohorts. Significant genes for each of the sub-cohorts were derived from the union of the three sets of 100 significant genes (derived from three classification tasks) for each of the correctly predicted samples. For example, 984 (4 + 1 + 2 + 6 + 3 + 4 + 88 + 876) significant genes for the LUAD-AAM sub-cohort were derived from the union of three sets of 100 significant genes for each of 18 correctly predicted samples (Table 3). Similarly, 1244 significant genes for the LUAD-EAM sub-cohort were derived from the union of significant gene sets for 129 correctly predicted samples.

**Genes common among sub-cohorts:** Figure 3a reveals that a large proportion of genes (876) are common across all sub-cohorts in LUAD, highlighting common molecular features that are preserved despite demographic differences. These genes potentially capture shared biological processes and microenvironmental influences contributing to the common behavior of LUAD cohorts. Interestingly, we also observed several shared gene sets across multiple sub-cohorts. Notably, 116 genes are shared among AAF, EAM, and EAF, while 88 genes are common to AAM, EAM, and EAF, suggesting the presence of conserved biological processes that may play a significant role in LUAD pathogenesis. Similar behavior is noticed across sub-cohorts in LUSC.

**Genes unique to individual sub-cohorts:** Figure 3a also highlights genes that are unique to individual sub-cohorts, suggesting that cohort-specific genetic variability may contribute to health disparities. For instance, only 4 genes are unique to the AAM sub-cohort. Similarly, 5, 29, and 50 genes are uniquely associated with the AAF, EAM, and EAF sub-cohorts, respectively. These sub-cohort-specific gene sets warrant further investigation to better understand the molecular basis of disparity across demographic groups. These findings confirm the ability of our method to identify both sub-cohort-specific and shared gene sets, reinforcing the need for disparity-aware models in the identification of clinically actionable biomarkers and therapeutic targets.

A similar pattern is observed in the LUSC cohort, as shown in Figure 3b, where sub-cohort-specific and shared gene sets demonstrate analogous trends of molecular divergence and overlap across demographic sub-cohorts. These findings reinforce the generalizability of our proposed approach. The lists of unique genes for each sub-cohort in both LUAD and LUSC are presented in Table 4.

**Figure 4:**
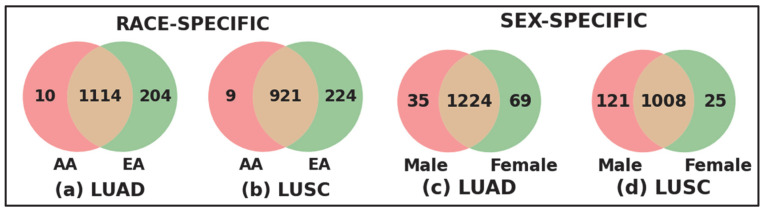
Race-specific and sex-specific gene set overlaps in LUAD and LUSC cohorts. The Venn diagrams compare the SHAP-derived significant genes between African American (AA) and European American (EA) patients for LUAD and LUSC cohort shown in (**a**,**b**), and between male (M) and female (F) sub-cohorts for LUAD and LUSC shown in (**c**,**d**). The large number of shared genes indicates common characteristics across populations, while the sub-cohort-specific unique genes highlight race- and sex-specific gene level disparities.

**Race- and Sex-specific disparity:** To further investigate the race- and sex-specific disparity patterns in LUAD and LUSC, we aggregated genes from four demographic sub-cohorts, AAM, AAF, EAM, and EAF, into broader racial and sex-based categories shown in Figure 4. For example, the African American (AA) cohort was defined as the union of AAM and AAF, and the European American (EA) cohort combined EAM and EAF (Figure 4a for LUAD and Figure 4b for LUSC). Similarly, the Male (M) group consists of AAM and EAM, and the Female (F) group includes AAF and EAF (Figure 4c for LUAD and Figure 4d for LUSC). Venn diagrams were constructed to compare the overlap and uniqueness of gene sets between these categories. As shown in Figure 4, both LUAD and LUSC exhibited not only considerable overlap between race- and sex-defined groups but also retained distinct sets of genes unique to AA, EA, Male, and Female cohorts. These unique gene subsets highlight potential molecular drivers of disparity that are specific to race and sex, warranting further functional investigation.

### 3.5. Recovery of Pathway-Level Disparity Within LUAD and LUSC Cohort

**Figure 5:**
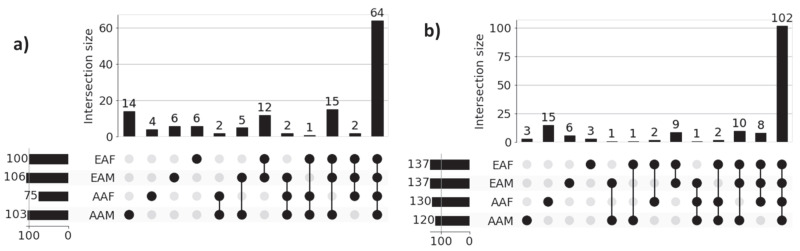
UpSet plots of sub-cohort-specific enriched pathways in (**a**) LUAD and (**b**) LUSC. Horizontal bars in the plot represent sub-cohort-specific significant pathways. For LUAD, these numbers are—AAM: 103, AAF: 75, EAM: 106, and EAF: 100. First 4 vertical bars represent the number of unique significant pathways for each sub-cohort, and the remaining bars represent the overlapping significant pathways between sub-cohorts. Most of the sub-cohort pathways are common in both LUAD (64 pathways) and LUSC (102 pathways), indicating population-dependent pathway signatures. Only a limited number of pathways are unique to individual sub-cohort indicating distinct pathway signatures.

To investigate disparities at the pathway level across racial and sex sub-cohorts, we performed pathway enrichment on sub-cohort-specific genes within the LUAD and LUSC cohorts. Figure 5 presents the UpSet plots highlighting the intersections of significantly enriched pathways among four sub-cohorts. The functional enrichment analyses were performed on AAM, AAF, EAM, and EAF sub-cohort-specific genes 984, 1024, 1244, and 1279, respectively, shown in Figure 3a for LUAD samples. Similarly, 850, 733, 1119, and 1024 genes were used for LUSC samples, shown in Figure 3b. Note that all the pathways in g:Profiler were used to identify the significant pathways based on *p*-value < 0.05.

Figure 5a presents the results of functional enrichment analysis for LUAD sub-cohorts, while Figure 5b illustrates the corresponding analysis for LUSC sub-cohorts. In both cases, the most prominent intersections represent pathways commonly enriched across all four demographic groups, reflecting a core set of shared biological processes within each cancer type. However, the presence of smaller, non-overlapping intersections highlights sub-cohort-specific pathway enrichment patterns, indicative of molecular heterogeneity across race and gender. For instance, within the LUAD cohort, the AAM, AAF, EAM, and EAF sub-cohorts exhibit 14, 4, 6, and 6 unique enriched pathways, respectively. Similarly, in the LUSC cohort, the AAM, AAF, EAM, and EAF groups show 3, 15, 6, and 3 unique enriched pathways, respectively. These findings emphasize the shared and unique transcriptional factors in each sub-cohort that may contribute to disparities in disease mechanisms and therapeutic outcomes. The names of the sub-cohort-specific unique pathways are listed in Table 5.

**Literature Validation:** Among the 14, 4, 6, and 6 significant pathways identified for the LUAD sub-cohorts AAM, AAF, EAM, and EAF, respectively, 9, 4, 3, and 4 pathways are found in existing literature as being associated with LUAD. Similarly, for the LUSC cohort, 2 out of 3 (AAM), 8 out of 15 (AAF), 3 out of 6 (EAM), and 1 out of 3 (EAF) significant pathways are found in the literature related to LUSC. These findings, supported by both statistical significance and external validation, highlight the biological relevance of the identified sub-cohort-specific pathways and underscore the effectiveness of our proposed XAI-based approach in uncovering meaningful disparities in lung cancer.

### 3.6. Generalizability

**Figure 6:**
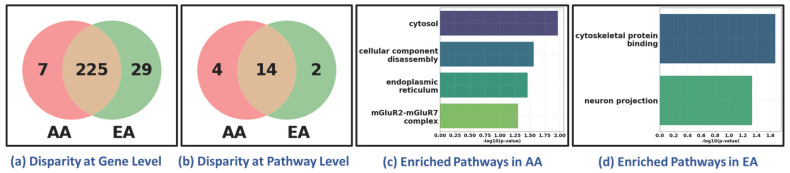
Health Disparity in Breast Cancer (BRCA). Disparity at (**a**) gene level and (**b**) pathway level. Unique (**c**) AA-specific and (**d**) EA-specific enriched pathways. AA: African American; EA: European American. The Venn diagrams in (**a**,**b**) show that while most genes and pathways are shared between AA and EA cohorts, a subset remains unique to each group, reflecting sub-cohort-specific molecular features. The bar plots in (**c**,**d**) highlight distinct enriched pathways in AA and EA patients, suggesting ancestry-related biological variation.

To evaluate the generalizability of our framework in discovering health disparities, we conducted an additional experiment using breast cancer data, focusing exclusively on female samples. The classification task was designed to distinguish two disease states (tumor vs. healthy). Tumor (*n* = 711) and healthy samples (*n* = 89) were obtained from TCGA BRCA and GTEx normal breast tissue, respectively. We used the same classifier, XGBoost, for classification, achieving 99.63% accuracy and correctly distinguishing 710 BRCA tumor samples from 87 healthy controls. We further analyzed its performance across racial sub-cohorts to assess the framework’s ability to delineate the disparities between African American (AA, 129 samples) and European American (EA, 582 samples). Despite the imbalance in sample size (AA:EA ≈ 1:5), the classifier correctly predicted all 129 AA and 581 EA samples, demonstrating its generalizability across demographically imbalanced cohorts. Following the procedure to discover disparity as outlined in the previous sections, we observed molecular-level disparities between AA and EA sub-cohorts, as shown in Figure 6. The disparity was evidenced at both the gene and pathway levels (Figure 6a,b). Bar plots of uniquely enriched pathways in AA and EA cohorts (Figure 6c,d) illustrate distinct biological processes reflecting disparity between AA and EA.

## 4. Discussion

The results presented in this study collectively demonstrate that the objectives of the proposed TILDA-X framework were successfully achieved. By designing classification problems based on disease conditions (LUAD, LUSC, and HEALTHY) instead of race, the framework effectively mitigated the imbalanced dataset issue in classification, achieving consistently high accuracy across all sub-cohorts. The bottom-up strategy, beginning from patient-specific SHAP interpretations and aggregating toward sub-cohort-specific patterns, successfully uncovered biologically meaningful biomarkers and pathways. These results validate our core hypothesis that individual-level interpretability can be leveraged to reveal population-level disparity signatures.

Importantly, the identified gene and pathway signatures showed substantial overlap with existing lung cancer literature, confirming the biological validation of our findings. The concordance between our explainable AI-derived results and validated pathways in previous studies provides compelling evidence that the framework captures true biological variation. This overlap supports the use of interpretable machine learning as a trustworthy and biologically grounded approach for health disparity research.

In the following subsections, we summarize the known biological processes in tumor development reported in the literature for the identified pathways for LUAD and LUSC in each of the sub-cohorts (AAM, AAF, EAM, and EAF) (see pathways identified with asterisk in Table 5).

**LUAD AAM Sub-cohort:** Disruption of anchoring junctions undermines epithelial barrier integrity, promotes loss of polarity, and facilitates invasion and metastasis in NSCLC, with altered claudins/occludins frequently implicated in lung tumors and lung metastasis [38]. Cancer-associated hypercoagulability contributes to tumor progression and poor outcomes in lung cancer, with meta-analytic evidence of elevated D-dimer and fibrinogen and other coagulation abnormalities in patients [39]. Cytoskeleton reorganization fuels EMT, motility, and metastasis, exemplified by BACH1-driven metastatic programs in LUAD and broader links between actin stress fibers and invasive phenotypes [40]. The AP-2B (TFAP2B) transcription factor is overexpressed in LUAD and drives tumor growth via ERK and VEGF/PEDF signaling, associating with poor prognosis and plausibly regulating target motifs consistent with AP-2 binding [41]. Tumor cells intensify biosynthetic output and macromolecule metabolism, lipid/mevalonate, glycolytic, glutaminolytic, and mitochondrial programs, to support proliferation, survival, and redox balance in LUAD, supported by pathway-level and proteomic studies and systems models linking phosphorylation/acetylation/ubiquitination networks to metabolic rewiring in lung cancer [42,43,44,45,46]. Concordantly, protein/metabolic-process–based gene signatures stratify LUAD prognosis, underscoring clinical relevance of metabolic pathway activation [47]. Disease activity is mirrored in body fluids, e.g., urine proteome changes track lung adenocarcinoma progression, supporting the “regulation of body fluid levels” axis as a window into tumor biology and biomarker discovery [48]. Canonical developmental signaling (Notch, Hedgehog, Wnt, ErbB) orchestrates differentiation, proliferation, and tissue architecture, and transcriptomic dissection shows these networks differentially regulate LUAD vs. LUSC, implicating broad “regulation of cellular component organization” programs that reshape tumor structure and microenvironment [49].

**LUAD EAM Sub-cohort:** Altered antiporter activity, especially the cystine/glutamate antiporter SLC7A11, supports redox homeostasis and therapy resistance, linking transporter up-regulation to tumor growth and metastatic potential in NSCLC [50,51]. Cytoplasmic vesicle pathways reflect the central role of extracellular vesicles (EVs) in lung cancer communication, where tumor- and TME-derived EV cargo promotes stemness, invasion, immune evasion, and offers biomarker/therapeutic opportunities [52,53]. Defense-response programs capture the known importance of innate/adaptive immunity in NSCLC pathogenesis and immunotherapy response [54]. Developmental growth/morphogenesis pathways are repeatedly co-opted in lung tumors, consistent with PTEN and growth-factor control of lung branching morphogenesis and with Notch/Hedgehog/Wnt/ErbB signaling differences between LUAD and LUSC [49,55]. The ZNF253 transcription factor shows cancer-tissue expression including lung, supporting a putative regulatory axis consistent with its reported motif activity in tumors [56]. Golgi-apparatus dysregulation yields prognostic signatures in LUAD and represents an actionable organelle target influencing protein processing and therapy response [57]. Lipid localization and transport terms align with mounting evidence that lipidomic remodeling shapes LUAD biology and immunotherapy efficacy, with plasma lipid signatures distinguishing LUAD and lipid-metabolism modulation improving anti-PD-1 outcomes [58]. Heightened demand for nucleotide precursors in proliferating tumors explains enrichment of nucleobase-containing small-molecule and nucleoside biosynthetic processes; in LUAD, enhanced purine nucleoside biosynthesis correlates with worse survival [59,60]. Organic-hydroxy compound transport captures lactate shuttling via monocarboxylate transporters (MCT1/MCT4), a hallmark of Warburg-driven NSCLC that fuels invasion and portends poor prognosis [61,62,63]. Secondary active transmembrane transporter activity, dominated by SLC families, drives nutrient uptake, drug response, and metabolic plasticity and is increasingly viewed as a therapeutic vulnerability across cancers including lung [64]. Enrichment of small-molecule biosynthetic programs aligns with systems-level analyses in LUAD that highlight metabolic/biosynthetic pathway activation as central to tumor progression and druggability [65]. Supramolecular fiber organization underscores the invasion toolkit of lung tumors: leader cells assemble long, stable filopodia and lay fibronectin tracks that coordinate collective migration and metastasis [66].

**LUAD AAF Sub-cohort:** Muscle structure development reflects pathways linked to cancer-associated cachexia, a frequent and lethal comorbidity in advanced lung cancer, where systemic inflammation and tumor-derived factors promote skeletal muscle wasting, mitochondrial dysfunction, and altered myogenic signaling, collectively impairing patient survival and treatment tolerance [67]. On the metabolic side, purine nucleoside monophosphate catabolic, purine ribonucleoside metabolic, and purine ribonucleoside monophosphate catabolic processes are central to maintaining nucleotide balance in rapidly proliferating cancer cells. Lung cancer cells exhibit enhanced purine turnover through AMP (adenosine monophosphate), GMP (guanosine monophosphate), and IMP (inosine monophosphate) catabolic routes, fueling energy generation, DNA/RNA synthesis, and redox control under hypoxia or nutrient stress. Recent metabolomic profiling revealed that dysregulated purine metabolism supports NSCLC cell proliferation, drives immunosuppressive adenosine accumulation in the tumor microenvironment, and correlates with poor prognosis and resistance to targeted therapies [68].

**LUAD EAF Sub-cohort:** Aberrant cell growth is a hallmark of lung tumorigenesis, where dysregulation of oncogenic signaling cascades such as PI3K/AKT/mTOR and MAPK promotes uncontrolled proliferation and tumor expansion; recent evidence highlights that targeting these hyperactivated growth pathways can suppress tumor progression and enhance therapeutic efficacy in NSCLC [69]. Cell–cell signaling contributes to tumor development through intercellular communication between cancer cells and their microenvironment, particularly via cytokines, chemokines, and extracellular vesicles, which orchestrate angiogenesis, EMT, immune evasion, and metastatic dissemination in NSCLC [70]. The positive regulation of the growth pathway encompasses oncogenic programs that enhance proliferation and survival, as shown by the role of growth-promoting transcription factors and metabolic regulators that drive tumor expansion and therapy resistance in lung adenocarcinoma [71]. Finally, regulation of cell growth represents the dynamic balance between pro-growth and growth-inhibitory signals; disruption of this equilibrium, through deregulation of mTOR activity, oxidative stress responses, or transcriptional miscontrol, contributes to uncontrolled proliferation and tumor aggressiveness in NSCLC [72].

**LUSC AAM Sub-cohort:** The choline transmembrane transporter activity pathway is closely linked to lung cancer metabolism, as elevated choline uptake supports phospholipid synthesis, membrane remodeling, and oncogenic signaling. Overexpression of choline transporters and choline kinase α enhances NSCLC cell proliferation and correlates with poor prognosis, highlighting this pathway’s role in tumor growth and metabolic adaptation [73]. Meanwhile, the skin epidermis development pathway reflects dysregulated epithelial differentiation processes relevant to lung squamous cell carcinoma (LUSC), where genes controlling keratinization and epidermal structure (e.g., p63, Notch, Wnt) are frequently altered, promoting abnormal epithelial remodeling and tumor invasiveness [74].

**LUSC EAM Sub-cohort:** The enrichment of basal part of cell/basal plasma membrane terms aligns with the role of airway basal cells, stem/progenitor cells anchored to the basement membrane, as candidate cells-of-origin for lung squamous cell carcinoma; their heterogeneity, high DNA-damage tolerance, and clonal expansion during premalignant progression link basal-surface signaling and basement-membrane interactions to early tumorigenesis in the bronchial epithelium [75]. In parallel, mature B-cell differentiation involved in immune response is relevant because tumor-infiltrating B cells (including germinal center–like and plasma cell subsets within tertiary lymphoid structures) modulate antitumor immunity, correlate with prognosis, and represent emerging therapeutic targets in NSCLC [76].

**LUSC AAF Sub-cohort:** The activation of NIMA kinases (NEK9, NEK6, NEK7) pathway is closely linked to lung cancer cell cycle control, as these kinases regulate mitotic spindle formation and chromosomal stability, and their overexpression promotes proliferation, invasion, and poor prognosis in NSCLC [77]. The circulatory system process pathway relates to tumor angiogenesis and vascular remodeling, which supply nutrients and enable metastasis—key features of lung cancer progression [78]. Dysregulation of the Golgi apparatus affects protein trafficking and secretion; recent studies show that Golgi structural proteins like GM130 and GOLM1 modulate EGFR signaling and influence NSCLC progression and prognosis [79]. Similarly, mitotic nuclear division reflects unchecked proliferation driven by dysregulated mitosis and centrosome abnormalities, a hallmark of lung tumorigenesis [80]. The nitrogen compound transport pathway is essential for amino acid and nucleotide metabolism; lung tumors reprogram nitrogen flux to sustain biosynthesis and redox balance, promoting growth under hypoxic conditions [81]. Both positive regulation of growth and regulation of growth pathways capture oncogenic signaling (e.g., EGFR, KRAS, and Hippo/YAP) that drive proliferation, metabolic adaptation, and immune evasion in lung cancer [82].

**LUSC EAF Sub-cohort:** The negative regulation of cell cycle process pathway is directly implicated in lung cancer progression, as disruption of normal cell-cycle checkpoints enables uncontrolled proliferation and genomic instability. In lung tumors, tumor suppressors are frequently inactivated, while oncogenic signaling overrides inhibitory controls, promoting cell-cycle progression despite DNA damage. Recent work has shown that targeting CDK4/6-mediated checkpoints restores this negative regulatory control and enhances anti-tumor immunity in NSCLC, offering a promising therapeutic strategy [83].

## 5. Conclusions

This study presents TILDA-X, an XAI-based computational framework designed to uncover health disparities in lung cancer across race- and sex-specific patient cohorts. By structuring multiple classification problems centered on disease conditions rather than race labels, the proposed approach enables more robust and unbiased discovery of disparity-related signals. Classification tasks based solely on racial labels often struggle due to significant class imbalance, limiting their effectiveness in capturing race-specific molecular patterns. In contrast, our disease-condition-focused design, incorporating LUAD, LUSC, and HEALTHY controls as class labels, proves to be an innovative strategy that improves model performance across all sub-cohorts. Notably, TILDA-X achieved high classification accuracy for both African American and European American patients, overcoming the data imbalance issue due to race.

The identification of sub-cohort-specific significant genes and pathways by the proposed framework provides critical insights into the health disparities in LUAD and LUSC. These molecular differences, revealed through explainable AI and pathway enrichment, suggest that disease progression and therapeutic responses may vary substantially between demographic sub-cohorts. The broader implication of this finding is the potential to guide precision medicine strategies that are tailored not only to disease subtype, but also to patient demographics. This study lays the foundation for developing sub-cohort-aware biomarkers, designing demographic-specific therapeutic interventions, and informing clinical trial stratification to reduce disparities in treatment outcomes.

This study is based on transcriptome data only. Thus, the findings call for deeper investigation into the biological mechanisms driving these disparities, including integration with genomic, epigenomic, proteomic, and immunologic data. Future work should focus on multi-omics integration to uncover the broader regulatory landscape contributing to demographic-specific tumor behavior. In parallel, functional validation of the identified sub-cohort-specific genes and pathways through in vitro and in vivo studies will be crucial to confirm their biological relevance. Lastly, collaboration with public health and policy stakeholders will be essential to translate these molecular insights into actionable interventions aimed at reducing lung cancer disparities in real-world clinical settings.

## Figures and Tables

**Table 1:** Data Distribution Of LUAD, LUSC, And Healthy Samples. This table summarizes the total number of tumor and healthy samples used in this study, showing the representation of African American and European American cohorts across LUAD and LUSC disease types. AA: African American; EA: European American.

Disease	Cohort		Sample
LUAD	AA	EA	
Male	20	136	156
Female	27	173	200
**LUSC**			
Male	16	195	211
Female	12	72	84
**GTEX Healthy Samples**			**313**
**Total Samples**			**964**

**Table 2:** Accuracies of Machine Learning Algorithms in Four Classification Problems. This table compares the performance of multiple classifiers for LUAD–LUSC–HEALTHY, LUAD–LUSC, LUAD–HEALTHY, and LUSC–HEALTHY classification tasks. XGBoost consistently achieved the highest accuracy across all settings.

Classification Problems	Using Default Values of Hyperparameters						Tuned
	FCN	LR	NB	SVM	RF	XGBoost	XGBoost
LUAD-LUSC-HEALTHY	93.36 ± 2.67	94.60 ± 0.84	65.86 ± 13.29	40.77 ± 7.60	95.12 ± 1.48	96.16 ± 1.29	96.37 ± 1.57
LUAD-LUSC	94.01 ± 1.64	93.24 ± 0.57	92.33 ± 1.26	93.24 ± 1.48	94.62 ± 1.29	94.77 ± 1.49	95.24 ± 0.90
LUAD-HEALTHY	99.25 ± 0.67	99.25 ± 0.82	75.78 ± 9.68	58.59 ± 10.63	98.96 ± 0.60	99.10 ± 0.56	99.25 ± 0.47
LUSC-HEALTHY	99.01 ± 0.96	99.18 ± 0.52	73.88 ± 11.59	56.77 ± 10.46	99.51 ± 0.66	99.51 ± 0.40	99.51 ± 0.40

**Table 3:** Race- and Sex-Specific Prediction using Different Approaches. This table presents the classification accuracies of three different methods for predicting race- and sex-specific sub-cohorts across LUAD and LUSC. The proposed framework, TILDA-X, achieved notably higher accuracies for underrepresented groups such as African American males (AAMs) and females (AAFs). These results demonstrate that the proposed approach effectively mitigates class imbalance issue due to race.

	LUAD Cohort				LUSC Cohort			
Sub-Cohort	Actual	Correct Prediction			Actual	Correct Prediction		
		Proposed	4-Class: LUAD	8-Class		Proposed	4-Class: LUSC	8-Class
AAM	20	18 (90%)	0 (0%)	0 (0%)	16	14 (88%)	1 (6%)	1 (6%)
AAF	27	26 (96%)	2 (7%)	2 (7%)	12	12 (100%)	0 (0%)	2 (16%)
EAM	136	129 (95%)	133 (98%)	127(93%)	195	182 (93%)	194 (99%)	184 (94%)
EAF	173	161 (93%)	172 (99%)	165 (95%)	72	63 (88%)	71 (87%)	63 (88%)
Total	356	334 (94%)	307 (86%)	294 (83%)	295	271 (92%)	266 (90%)	250 (85%)

**Table 4:** Significant Unique Genes of LUAD and LUSC Sub-cohorts. The table lists the genes uniquely identified for each sub-cohort within LUAD and LUSC based on SHAP scores. The presence of distinct gene sets across sub-cohorts (AAM, AAF, EAM, and EAF) highlights the existence of race- and sex-specific gene-level disparities.

Sub-Cohort	LUAD	LUSC
AAM	CPT2, ETHE1, TTLL2, XRCC3	CCL4L2, DNLZ, MAP1LC3A, MLKL, NMRK2, SHKBP1
EAM	ABCB5, CRYBA1, PRKX, RP11-351M8.1, SYCE1L	RAB40A, SUPT5H
AAF	AMT, APOBEC3G, ARG2, CIDEC, CMPK2, CYBA, CYP3A5, EIF2AK1, ERVW-1, FUT3, GOLGA8J, MAPK10, MBOAT4, MS4A18, MTHFD1L, NMUR1, OR7A5, PAK2, PEX5, POU2F1, RP11-849F2.7, SLC26A9, SLC32A1, SLC8A2, SMIM7, TNFAIP8L2, TRPS1, ZMYND8, ZNF324B	ADCYAP1, AGPAT4, ANGEL1, APOE, ARG2, ATP2B2, AURKAIP1, BEND5, C10orf10, C10orf90, C12orf40, C16orf74, CAMKK2, CASP1, CASP5, CCAR2, CCDC12, CD276, CD58, CDC42BPA, CELA3A, CEP170B, CPAMD8, CSRP2, CT45A4, CTD-2287O16.3, CXCL3, CYP24A1, DBH, DDX3Y, DMC1, DTYMK, EFR3B, ETHE1, EVC2, F2, FA2H, FAM222B, FER, GPR113, GRIA2, ID1, ITPKB, KRT72, KRTAP13-1, LDB3, LINGO3, LPHN3, MAN1B1, MB21D2, MFAP3, MFAP4, MGRN1, MRPL14, MRPL53, MTFR1, NDST3, NKAPL, NMNAT2, NPIPA5, NR3C1, NYAP1, OR8G5, P2RX5-TAX1BP3, PDE6A, PDIA2, PGLYRP1, PIGW, POU2AF1, PPP1R37, PRAMEF12, PRRG4, PRSS3, PTK2, PXDN, RAB7L1, RAPGEF1, RDH14, RGPD2, RHBDD1, RP11-302B13.5, RP11-794P6.2, SAA4, SCEL, SERTAD1, SFT2D3, SLC26A7, SLC6A7, SMIM7, SNX7, TDRD15, TMEM101, TMEM25, TOR1A, TRIM36, TSHR, UPK1A, YARS, ZNF324B, ZNF514, ZNF766
EAF	AC135983.2, AGPAT4, AL139099.1, ALDOA, BAI1, BRF1, C10orf90, C18orf54, C6orf222, CAMKK2, CCDC168, CEACAM21, CELF5, CNTF, CSRP2, CXCL12, DPRX, DUT, F2, GPR150, GPR84, KCNIP4, KIAA0195, KRTAP13-1, LRRC8E, MAN1C1, MAPK15, MFAP3, MKI67, MYL5, MYO15B, NBPF11, NUTM2B, OR8G5, PRAC1, PSG2, RHBDD1, RP11-77K12.1, SAMD12, SLC1A4, SYN1, TEX101, TLDC1, TMEM130, TMEM17, TMEM25, ZFAND5, ZNF219, ZNF337, ZNF436	AC026740.1, AHNAK2, C18orf54, C6orf203, CAMSAP2, CD244, DLG5, EDEM1, FBXO11, G6PC2, GATAD1, GFPT2, GPR84, ISM2, KCNK4, LRRC8E, OCSTAMP, PRLH, SBSPON, SKP2, TFDP3, TMEM156

**Table 5:** Significant Unique Pathways of LUAD and LUSC Sub-cohorts. Asterix (*) indicates pathways reported in existing literature. This table summarizes the biologically significant pathways uniquely enriched within each race and sex sub-cohort of LUAD and LUSC. The distinct pathway patterns observed across sub-cohorts (AAM, AAF, EAM, and EAF) reveal sub-cohort-specific disparities at pathway level.

Sub-Cohort	LUAD	LUSC
AAM	* Anchoring Junction, * Blood Coagulation, * Cytoskeleton Organization, * Factor: AP2BETA; Motif: SCCYCAGGSNN, Formation of The Cornified Envelope, Hindlimb Morphogenesis, Kidney; Distal Tubules [≥low], * Positive Regulation of Cellular Biosynthetic Process, * Positive Regulation of Macromolecule Biosynthetic Process, * Positive Regulation of Macromolecule Metabolic Process, * Protein Metabolic Process, * Regulation of Cellular Component Organization, Tissue Morphogenesis, Tube Morphogenesis	* Choline Transmembrane Transporter Activity, Pancreatic Cancer Subtypes, * Skin Epidermis Development
EAM	* Muscle Structure Development, * Purine Nucleoside Monophosphate Catabolic Process, * Purine Ribonucleoside Metabolic Process, * Purine Ribonucleoside Monophosphate Catabolic Process	* Activation of Nima Kinases Nek9, Nek6, Nek7, * Axon, * Circulatory System Process, Developmental Biology, * Golgi Apparatus, Macromolecule Localization, * Mitotic Nuclear Division, Negative Regulation of Chromosome Organization, * Nitrogen Compound Transport, * Positive Regulation of Growth, Positive Regulation of Multicellular Organismal Process, Regulation of Anatomical Structure Size, Regulation of Cellular Component Organization, * Regulation of Growth, Regulation of Organelle Organization
AAF	* Antiporter Activity, Defense Response, * Lipid Transport, Regulation of Response to Stimulus, Response to External Stimulus, * Small Molecule Biosynthetic Process	* Basal Part of Cell, * Basal Plasma Membrane, Basolateral Plasma Membrane, Factor: cpbp; Motif: SNCCCNN; Match Class: 1, * Mature B Cell Differentiation Involved in Immune Response, Response to Stress
EAF	* Cell Growth, * Cell–cell Signaling, Factor: ZNF253; Motif: SNGNSCGNGGNGCKGNN; Match Class: 1, * Positive Regulation of Growth, Regulation of Anatomical Structure Size, * Regulation of Cell Growth	* Negative Regulation of Cell Cycle Process, RNA Polymerase II-specific DNA Transcription Factor Binding, Skin 2; Cells in Corneal Layer [≥low]

## Data Availability

The original data presented in the study are openly available in https://github.com/mskcc/RNAseqDB (accessed on 1 August 2024). The code is available in https://github.com/codebysobhan/HealthDisparity (accessed on 1 August 2024).
